# Delay in Diagnosis of Autoimmune Polyendocrine Syndrome Type 2 as a Consequence of Misinterpretation of Gastrointestinal Symptoms

**DOI:** 10.1155/2022/6623020

**Published:** 2022-03-23

**Authors:** Maciej Gonciarz, Michał Krogulecki, Dorota Brodowska-Kania, Szczepan Cierniak, Grzegorz Kamiński

**Affiliations:** ^1^Military Institute of Medicine in Warsaw, Department of Gastroenterology and Internal Medicine, Warsaw, Poland; ^2^Military Institute of Medicine in Warsaw, Department of Endocrinology and Radioisotope Therapy, Warsaw, Poland; ^3^Military Institute of Medicine in Warsaw, Department of Pathology, Warsaw, Poland

## Abstract

**Background:**

Type 2 autoimmune polyendocrine syndrome (APS-2) is characterized by the presence of at least two of three endocrinopathies: Addison's disease, autoimmune thyroiditis, and diabetes type 1. The prevalence of APS-2 is estimated to be 1 : 1000 to 1 : 20.000 in the general population. Diagnosis of APS-2 often is delayed due to its rarity and wide spectrum of clinical symptoms. *Case Presentation*. A 27-year-old presented with a 6-month history of abdominal pain, vomiting, diarrhea, weakness, fatigue, and 15 kg of weight loss. The patient was diagnosed with Crohn's disease in a local hospital and referred to our institution because of treatment failure. Colonoscopy performed in this hospital identified irregular mucosal erosions in terminal ileum, and the microscopy of biopsy specimens demonstrated nonspecific inflammation. On physical examination, the patient appeared cachectic. Blood pressure was 90/60 mmHg. Laboratory results were significant for severe hyponatremia and mild hyperkalemia. Morning cortisol was low, and adrenocorticotropic hormone (ACTH) concentration was high. An ACTH stimulation test did not present any increase in serum cortisol, which confirmed primary adrenal insufficiency. Antithyroid peroxidase antibody (anti-TPO) as well as both anti-21-hydroxylase antibodies and antiglutamic acid decarboxylase antibodies (GAD65) were positive. So, the diagnosis of APS-2 was made, and the replacement doses of hydrocortisone and fludrocortisone has brought a rapid improvement in all clinical symptoms; colonoscopy showed normal.

**Conclusion:**

The case presented herein highlights rapidly progressive nature of untreated APS-2 and that the diagnosis of APS-2 may be challenging.

## 1. Background

Autoimmune polyendocrine syndromes (APSs) also named polyglandular deficiency syndromes are rare disorders characterized by functional impairment of multiple endocrine glands due to loss of immune [1]. Three different clinical presentations can be distinguished:

- A very rare monogenic autoimmune polyendocrine syndrome type 1 (APS-1) that affects approximately 1 in 80 000 individuals in the general population. It is juvenile type inherited in an autosomal recessive manner and defined by the presence of 2 or more of the following: chronic mucocutaneous candidiasis, hypoparathyroidism, and adrenal insufficiency. Symptoms usually occur in young patients; however, other endocrine and nonendocrine disorders may appear until the age of 40 [1].
- A more common polygenic variety, autoimmune polyendocrine syndrome type 2 (APS-2) or Schmidt's syndrome named after Schmidt who originally described 2 cases of Addison disease and chronic lymphocytic thyroiditis in 1926 [2]. According to recent epidemiological data, the prevalence of APS-2 is estimated to be 1 : 1000 to 1 : 20000 in the general population [1]. APS-2 consists of Addison's disease, autoimmune thyroiditis, and/or diabetes type 1. At least two of these endocrinopathies are of diagnostic value. The complete triad of Addison's disease, autoimmune thyroiditis, and type 1 diabetes is termed Carpenter's syndrome [3]. APS-2 usually occurs in adults and more often affects women. It may occur alone or in association with other autoimmune disorders such as celiac disease, alopecia, vitiligo, premature ovarian insufficiency, pernicious anemia, and others [1].
- Type 3 autoimmune polyendocrine syndrome (APS-3) includes autoimmune thyroiditis and other autoimmune diseases except for Addison's disease [4].

A careful management of specific disorders can prevent complications; however, because of the wide spectrum of clinical symptoms of APS and its rarity, the diagnosis often is delayed for months resulting in errors in patient management.

## 2. Case Report

**Figure 1:**
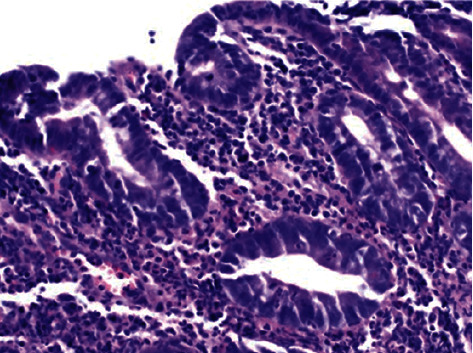
Ileum: the tissues are heavily infiltrated by extremely large quantities of inflammatory cells (transmural inflammation, lymphocytes and plasma cells). Hematoxylin-eosin, original magnification x 400.

In June 2019, a 27-year-old patient was admitted to our department due to abdominal pain, vomiting, diarrhea, weakness, fatigue, and 15 kg of weight loss, all of 6 month's duration. In November 2018, when he began to experience a weakness and fatigue, complained of abdominal pain, and multiple loose nonbloody bowel movements per day, he was referred to a tertiary hospital's outpatient department. Basic laboratory testing revealed only slight hyponatremia. Other laboratory tests such as antibodies, tissue transglutaminase IgA (tTG-IgA), *Saccharomyces cerevisiae* (ASCA), and perinuclear antineutrophil (pANCA) antibodies were all negative. Stool samples for parasites, clostridium difficile toxins, occult blood, and calprotectin were also negative. There were no significant deviations from the normal state in the chest X-ray, ultrasound, and upper gastrointestinal endoscopy examinations. The colonoscopy identified irregular mucosal erosions in terminal ileum, and the microscopy of multiple biopsy specimens demonstrated nonspecific inflammation. Though the histopathological reassessment revealed heavy infiltration by extremely large quantities of lymphocytes and plasma cells (transmural inflammation) (Figure 1), a diagnosis of Crohn disease was also made. The patient was managed with budesonide 9 mg/day and mesalamine at a dose of 2.0 g/day. After two months, due to a result of treatment failure, azathioprine 100 mg/day was initiated. However, his bowel symptoms did not improve, while weakness and fatigue gradually aggravated, and the patient lost 15 kilograms of his weight. At this point, he was referred to our institution for further management.

His personal and family history was unremarkable, while only Hashimoto disease was recognized in the patient's mother and sister. On physical examination, the patient appeared cachectic (56 kg, 165 cm), his epigastrium was tender, and the palms were with slightly increased pigmentation. Blood pressure was 90/60 mmHg, and heart rate was 100 beats/min. Otherwise, the physical examination was unremarkable. An ultrasound study of the abdomen and MR enterography were normal.

Laboratory results were significant for severe hyponatremia-sodium 119 mmol/l (reference: 135–145 mmol/l) and mild hyperkalemia-potassium 5.5 mmol/l (reference: 3.5–5.0 mmol/l). Laboratory tests showed the following results: the chloride concentration: 84 mmol/l (reference: 95–106 mmol/l), pH: 7.353, HCO_3_^−^: 21.3 mmol/l (reference: 22–29 mmol/l), and glucose: 4.38 mmol/l (reference: 3.9–5.5 mmol/l). Other biochemical tests including blood count, creatinine, and urea revealed no abnormalities. A morning cortisol was low at 6.63 *µ*g/dl (reference: >10 *µ*g/dl), and adrenocorticotropic hormone (ACTH) concentration was high at 1662 pmol/l (normal: 7.2–63.3 pmol/l). An ACTH stimulation test (short Synacthen (tetracosactide—a peptide representing the sequence of the first 24 (out of a total of 39)) amino acids of ACTH) did not present any increase in serum cortisol, which confirmed primary adrenal insufficiency. The tuberculin test and interferon-gamma release assay (IGRA) were negative. Dynamic contrast-enhanced magnetic resonance of the pituitary gland showed normal. We did not observe any other laboratory abnormalities (ANA, SM, ANCA, anti-LKM1, anti tGT-IgA, fT3, fT4, TSH, anti-TPO, ICA, IAA, and ICSA).

Antiglutamic acid decarboxylase antibodies (GAD65) were positive in titer of 158.1 IU/ml (positive ≥10 IU/ml). Given those findings, the diagnosis of autoimmune polyglandular syndrome type 2 was made, and the patient was started with replacement doses of hydrocortisone (35 mg per day) and fludrocortisone (0.1 mg per day). A week 12 colonoscopy and multiple colonic biopsies showed no abnormalities. At the 24-week follow-up, no signs of hypocortisolemia were present. In the 75 mg oral glucose tolerance test (OGTT), there were normal glucose concentration before (5.27 mmol/l) and after 120 min (4.67 mmol/l), with signs of insulin resistance (inulin concentration too high, 51.8 *µ*U/ml, after 2 hours in OGGT). This points to a risk of developing type 1 diabetes. Thyroid function remains normal with TSH 1.39 uUI/ml, fT4: 21.8 pmol/l (range: 10.3–30.9 pmol/l), and fT3: 5.38 pmol/l (range: 2.25–6.0 pmol/l). Sex hormone concentration remains within normal range, with testosterone concentration of 4.11 ng/ml (range: 2.8–8.2 ng/ml), FSH: 10.74 IU/l (range: 1.5–12.4 IU/l), and LH: 4.62 IU/ml (range: 1.7–8.6 IU/ml). Serum ACTH concentration is only 2.5 times increased, 163.6 pmol/ml, with normal sodium (Na, 135 mmol/l) and potassium (K, 4.2 mmol/l) concentration, what proves a good correction of hypocortisolemia in this case.

## 3. Discussion

This case highlights the nonspecific, varied presentation, and rapidly progressive nature of untreated APS-2. Immediate diagnosis and appropriate management are essential for a good outcome. The clinical presentation of APS type 2 includes a wide range of severity, from mild to seriously impaired patients. In our case, the degree of clinical symptoms could be placed in the high range. Symptoms can often be difficult to distinguish from gastroenterological etiologies, resulting in inaccurate diagnosis. In our case, the diagnosis of Crohn's disease made prior to admission was misleading. CRP and stool calprotectin were normal, and histology of ileal lesions showed no crypt distortion, cryptitis, crypt abscess, or granuloma. Misdiagnosis resulted in errors in patient management. The patient treated with mesalamine, budesonide, and azathioprine continued to be symptomatic, while the colonoscopy performed three months later with bare replacement doses of hydrocortisone showed no abnormalities.

The first clinical case showing difficulties of different diagnosis, Addison's and Crohn's disease, was described in 1958 [5]. All patients have gastrointestinal symptoms in Addison's disease. Usually, diagnosis is made with a delayed average at 6 months. The effect of this delay is an adrenal crisis, which it was in this case. A common feature of Addison's disease and Crohn's disease is progressive cachexia, anorexia, weight loss, vomiting, diarrhea, abdominal pain, depression, arthritis symptoms, and response to oral corticosteroids [6]. In Addison's disease, the most typical symptoms are hyperpigmentation, postural drop, muscle weakness, sex hormone, and electrolyte disturbances (hyponatremia and hyperkaliemia).

Our patient did not suffer from type 1 diabetes, but an antibody against GAD 65 for type 1 diabetes was positive with titers of 158.1 IU/ml. Other antibodies typical for type 1 diabetes were not detected. The presence of anti-TPO antibodies with a typical ultrasound image of the thyroid gland allows for an autoimmune thyroiditis diagnosis. However, the major clinical problem was severe hypoadrenocorticism associated with type 21-hydroxylase antibodies. Primary adrenal insufficiency, Addison's disease, is a life-threatening condition, so the early diagnosis and glucocorticoid therapy are crucial. In the case presented here, the delay in accurate diagnosis was about 6 months. The progressing autoimmune process resulted in adrenal crisis (hyponatremia, hyperkaliemia, hypotension, and hypoglycemia). Berkelhammer et al. [7] described a rare case of severe adrenal insufficiency complicating budesonide maintenance therapy for Crohn' disease. The authors cite the results of 11 studies which showed that patients with Crohn's disease on budesonide for maintenance of remission showed abnormal ACTH stimulation tests nearly three times more frequent than those of control groups. Our patient was on budesonide for several months before admission; thus, it could play an additional role in his adrenal dysfunction.

In this case, the coincidence of Addison's disease with autoimmune thyroiditis allowed for the diagnosis of the APS type 2 (Schmidt's syndrome). If diabetes appears, Carpenter's syndrome will be eligible [5, 6].

## Data Availability

The data used to support the findings of this study are included within the article and references therin.
